# Derivative Complex Small Supernumerary Marker Chromosomes (sSMC) Involving Chromosomes 2 and 15—A Novel Report

**DOI:** 10.1002/ccr3.70592

**Published:** 2025-07-30

**Authors:** Yazeed Alayed, Aziza Mushiba, Soha Tashkandi, Yahya Almashham, Khelad Alsaidi, Abdulrazaq Albohigan, Amer Jamhawi, Mohammad Alkheilewi, Maryam Alotaibi

**Affiliations:** ^1^ Department of Pediatric, Main Children Hospital King Fahad Medical City Riyadh Kingdom of Saudi Arabia; ^2^ Department of Pediatric Metabolic and Genetics, Main Children Hospital King Fahad Medical City Riyadh Kingdom of Saudi Arabia; ^3^ Cytogenetics Laboratory, Clinical Pathology, PCLMA King Fahad Medical City Riyadh Kingdom of Saudi Arabia; ^4^ Department of Pediatric Cardiology, Main Children Hospital King Fahad Medical City Riyadh Kingdom of Saudi Arabia; ^5^ Cytogenetics Laboratory, Main Children Hospital King Fahad Medical City Riyadh Kingdom of Saudi Arabia; ^6^ Eurofins Clinical Riyadh Kingdom of Saudi Arabia

**Keywords:** chromosomal microarray, congenital anomalies, duplication 15q, duplication 2p, neonate, supernumerary marker chromosomes

## Abstract

Small supernumerary marker chromosomes (sSMC) constitute a rare group of structural chromosomal abnormalities characterized by additional genetic material that cannot be identified by conventional banding cytogenetics. The incidence of sSMC is extremely rare, and most are expected to have no clinical phenotypic abnormalities. Advanced cytogenetic modalities are crucial for sSMCs identification, characterization, and analysis of chromosomal structure. An 8‐day‐old neonate born to a G2P2 mother with gestational diabetes and a history of infertility was admitted for respiratory distress. Clinical evaluations included chromosomal microarray, karyotyping, fluorescence in situ hybridization (FISH), brain magnetic resonance imaging (MRI), and cardiac computed tomography (CT). Initial echocardiography revealed atrial and ventricular septal defects, patent ductus arteriosus, and left pulmonary artery stenosis. Brain MRI showed trigonocephaly, ventriculomegaly, corpus callosum dysgenesis, and gray matter heterotopia. Chromosomal microarray identified proximal duplication of 15q and duplication of 2p. Despite intensive care and surgical interventions, the infant faced recurrent respiratory complications and failed extubation attempts. SMC involving chromosomes 15 and 2 presenting with multiple congenital anomalies delineate the genotype–phenotype correlation roadmap. Rapid and immense development in cytogenetics may expand further correlation strategies in the future.

## Graphical abstract

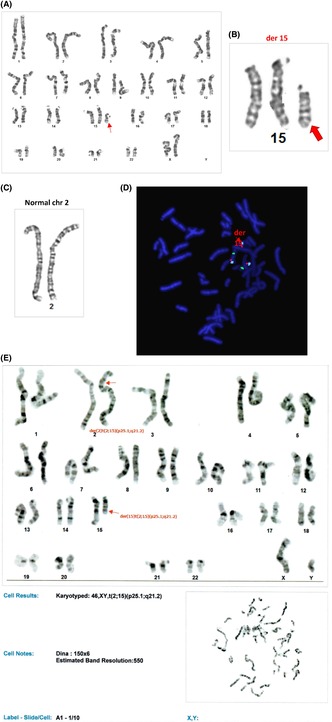

**Summary**

- The case report at hand describes a novel finding of sSMC involving chromosomes 15 and 2 in a female patient with dysmorphism and congenital brain anomalies.
- 3:1 segregation is observed resulting in the presence of normal homolog of chromosome 2 and normal homolog of chromosome 15 with additional paternally inherited derivative marker chromosome.

**Abbreviations**
ASD: atrial septal defect
CT: computed tomography
ES: Emanuel syndrome
FISH: fluorescence in situ hybridization
KFMC: to King Fahad Medical City
MRI: brain magnetic resonance imaging
PMVSD: perimembranous ventricular septal defect
sSMC: small supernumerary marker chromosomes

## 1 Introduction

Small supernumerary marker chromosomes (sSMC) constitute a rare group of structural chromosomal abnormalities characterized by additional genetic material that cannot be identified by conventional banding cytogenetic [1]. These chromosomes are typically small in size, often equal to or smaller than a chromosome 20 in the same metaphase spread. Cytogenetically, sSMCs are defined as structurally abnormal chromosomes that cannot be unambiguously identified or characterized using conventional banding techniques alone [2]. The incidence of sSMC is extremely rare, occurring in approximately 0.044% of newborns [3, 4]. Among those with sSMCs, 70% are expected to have no clinical phenotypic abnormalities [5]. These marker chromosomes can be derived from any of 24 chromosomes and can appear as either inverted duplication, ring, or centric minutes shaped derivative chromosomes [6]. Therefore, advanced cytogenetic modalities are crucial for sSMC identification, characterization, and analysis of chromosomal structure [7]. Furthermore, sSMCs can originate from a single chromosome, two chromosomes, or even more chromosomes; the latter entity constitutes the so‐called complex sSMCs [1, 8]. The clinical trajectory is typically more complicated if multiple marker chromosomes are involved [9]. Interestingly, the chromosome region 15q11q13 is known for its instability and is prone to genomic rearrangement [10].

In this case report, dual marker chromosomal aberrations, proximal duplication of 15q and distal duplication of 2p was diagnosed with a novel phenotype manifested by multiple congenital anomalies.

## 2 Case History and Examination

An 8‐day‐old full‐term female, with antenatal history of symmetrical intrauterine growth restriction born to a G2P2 mother with gestational diabetes and history of 10 years of infertility admitted to neonatal intensive care unit due to respiratory distress, wide pulse pressure and machinery heart murmur upon examination. Mode of delivery was via caesarean section due to fetal distress with a low birth weight of 1.95 kg. She was subsequently referred to King Fahad Medical City (KFMC) for further evaluation and management. Upon arrival to KFMC emergency department, she was noted to be lethargic, cyanosed and in respiratory distress with a significant decrease in oral intake. In the context of the initial worrisome wide pulse pressure and heart murmur, bedside echocardiography was performed yielding an initial diagnosis of atrial septal defect II (ASD), moderate ventricular septal defect, moderate patent ductus arteriosus, and left pulmonary artery origin stenosis.

Upon physical examination, the neonate was conscious but in mild respiratory distress and appeared dehydrated. A pansystolic murmur was appreciated in the left third parasternal intercostal space. Vital signs revealed a blood pressure of 89/56 mmHg, a heart rate of 174 beats per minute, and a respiratory rate of 64 breaths per minute. A chest x‐ray showed cardiomegaly, and an electrocardiogram demonstrated sinus tachycardia with a short PR interval. A follow‐up transthoracic echocardiogram showed moderate tricuspid regurgitation with a systemic gradient, a moderate ASD II with a fenestrated left‐to‐right shunt, moderate perimembranous ventricular septal defect (PMVSD) with a left‐to‐right shunt, accelerated flow in the right ventricular outflow tract with a pressure gradient of 21 mmHg, left pulmonary artery stenosis mostly at the origin with a pressure gradient of 65 mmHg, a small PDA with a left‐to‐right shunt, and normal left ventricular systolic function.

Admission ensued for stabilization. While she was under evaluation, she was transferred to pediatric intensive care due to increasing oxygen requirement and desaturation. Inotropic support was initiated for hemodynamic stability with proper sedation and started on antibiotics. Shortly thereafter, hypoxic hypercapnic respiratory failure emerged, and she was intubated and placed on mechanical ventilation.

## 3 Investigations, Differential Diagnoses, and Treatment

**FIGURE 1:**
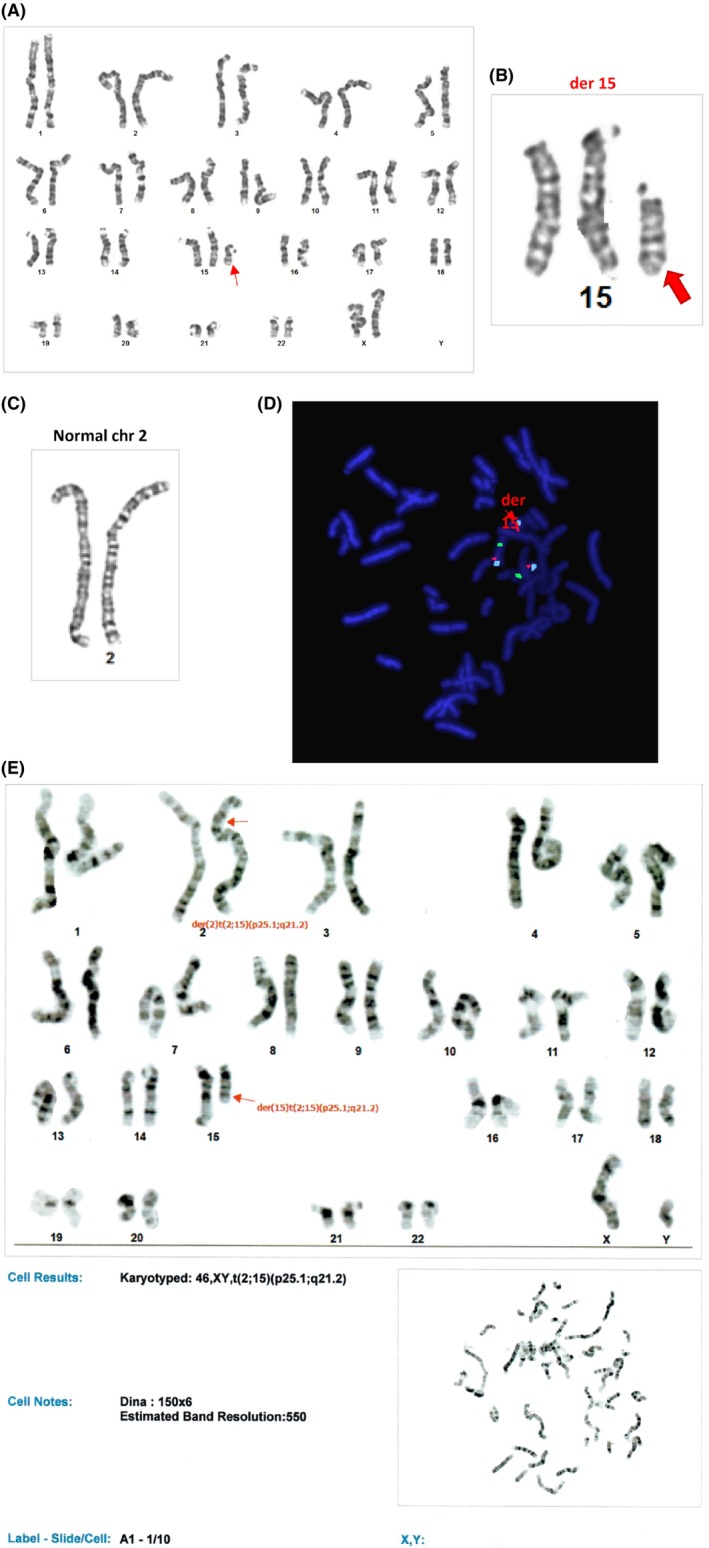
(A) Karyotyping of the patient. (B) Complex sSMC of chromosome 15 derivative. (C) Chromosome 2 depicted as normally derived. (D) FISH analysis using Probe: D15S10(SO), PML(SG), and CEP15(SA). (E) Father karyotyping.

Amid her clinical deterioration, a brain ultrasound revealed increased ventricular dilatation and partial corpus callosum dysgenesis, prompting involvement of the pediatric metabolic and genetics department. She was observed to have microcephaly, trigonocephaly, a small anterior fontanelle, overlapping patent coronal and sagittal sutures, along with micropthalmia, hypertelorism, low‐set ears, and a minimally anteriorly displaced anus associated with a small tag at the anterior anal verge. Chromosomal analysis confirmed a female karyotype with an imbalanced translocation between chromosome 2 at band 2p25.1 and chromosome 15 at band 15q21 (Figure 1A–C). A recommendation for chromosomal microarray testing was made, which depicted a complex chromosomal abnormality in the form of proximal duplication of 15q15q11.1q21.2 and distal duplication of 2p 2p25.3p25.1 (Table 1). FISH testing using different probes is shown in (Figure 1D). Father karyotyping is shown in Figure 1E.

**TABLE 1:** Microarray findings and interpretation.

CNV description	Size	Gene count	Classification	Chromosomal region	Interpretation
arr[GRCh37] 15q11.1q21.2(20001839_51340711)x3	31,339 Mb	425	Pathogenic	15q11.1q21.2	Proximal duplication of 15q
arr[GRCh37] 2p25.3p25.1(10587_10140981)x3	10,130 Mb	66	Pathogenic	2p25.3p25.1	Distal partial trisomy 2p

**FIGURE 2:**
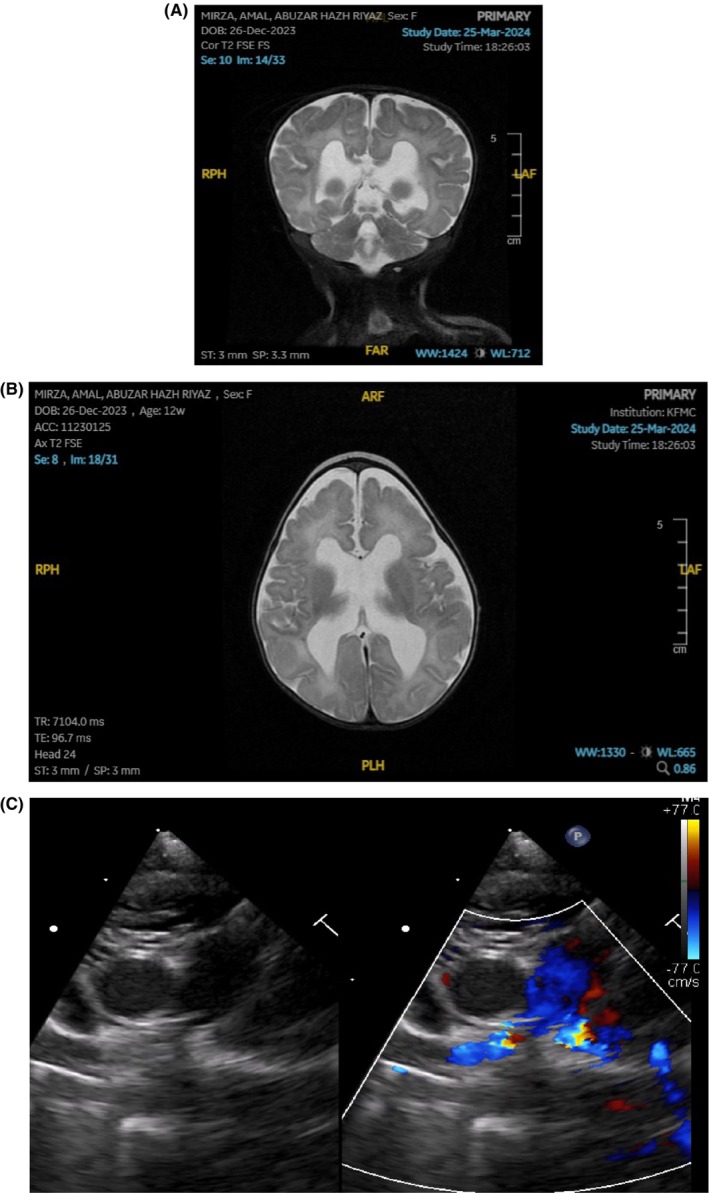
(A) Coronal view of T2 brain MRI. (B) Axial view of T2 brain MRI. (C) Echocardiographic image of the heart dynamics following insertion of right pulmonary artery flow regulator.

Furthermore, cardiac computed tomography of great vessels was performed revealing an ASD, VSD, and patent ductus arteriosus with an enlarged right atrium demonstrating retrograde flow of contrast to the inferior vena cava and hepatic veins indicating left pulmonary artery stenosis. Moreover, magnetic resonance imaging of the brain illustrates distinct trigonocephaly, partial premature fusion of metopic suture, moderately dilated ventricles, absence of septum pellucidum and stenosed aqueduct, prominent robust cerebrospinal fluid flow void in the mildly dilated prepontine cistern with likelihood of communication, dysplastic appearance of the fully formed and thin corpus callosum and hippocampal formation, gray matter heterotopia in the frontal horn, and the peritrigonal white matter as well as mildly increased T2 hyperintensity of the white matter in the supratentorial brain in keeping with mild edema (Figure 2A,B). Patient's phenotype is depicted in Image 1.

Cytogenetics testing was carried out on stimulated cultured lymphocytes from the newborn following the routine methods. In brief, two independent cultures were initiated from a peripheral blood sample in RPMI 1640; all culture additives are from Gibco Life Technologies, UK, except the FBS (PAA, Austria). Microscopic analysis for both karyotype analysis and FISH analysis was done using the Nikon 90i microscope (Nikon, Japan) and the Cytovision image analysis system (Applied imaging, USA). The fixed cell pellets were subjected to karyotype analysis on GTG‐banded chromosomes and FISH testing using the Prader‐Willi/Angelman syndromes probe panel, which included Vysis Prader‐Willi/Angelman Region LSI D15S11, LSI GABRB3, LSI SNRPN, and LSI D15S10, all probes from Abbott, USA. Microarray (CMA) testing was done at a reference laboratory, Centogene, for the CentoLCV test, and the sequences obtained are aligned to Genome Reference Consortium Human Build 37 (GRCh37/hg19).

## 4 Outcome and Follow‐Up

Multidisciplinary meeting was held to discuss medical and surgical management considering the disease prognosis. A decision to undergo right pulmonary flow regulator via cardiac catheterization was performed (Figure 2C). She failed extubation multiple times during her clinical course in spite of adequate respiratory and cardiac stabilization, rendering early demise.

## 5 Discussion

The abbreviation “sSMC” was formerly called “Marker chromosome” and designated as “mar” when writing the ISCN. Nowadays, they are referred to as “der” which stands for derivative chromosome. In Meiosis I, the four chromosomes with segments in common come together to form a quadrivalent. Spindles will then form at each pole of the cell to seek chromosomal attachment. The distribution of the four homolog segments of these chromosomes to each pole is a process referred to as segregation. In our case, 3:1 segregation is observed, resulting in the presence of a normal homolog of chromosome 2 and a normal homolog of chromosome 15 with an additional paternally inherited derivative marker chromosome (t:2:15). The mode of segregation is referred to as tertiary trisomy, which has led to two normal chromosomes of the quadrivalent segments plus one of the translocations going together to one daughter cell [11].

Beside Emanuel syndrome (ES), fewer than 100 cases of complex sSMCs have been reported. In addition, chromosome 15 has been described to be the most frequently affected chromosome [12]. The aforementioned statement was reflected by the involvement of chromosome 15 depicted in our case report. Insofar, this case report illustrates a unique and novel complex sSMC involving chromosomes 15 and 2 that has not been described before.

Around 70% of all sSMC have no distinct genotype–phenotype correlation in contrast to ES, cat eye syndrome, isochromosome‐18p, and Pillister Killian syndrome [13]. Almost half of sSMC carriers derived from chromosome 15 analyzed by Liher et al. [13] revealed a normal phenotype, a major culprit chromosome affected in our report. Only 22 cases involving chromosome 2 have been reported so far. Of whom, four were complex sSMC cases involving chromosome 2 suggesting extreme scarcity of our reported case. Therefore, the current report describes a novel phenotype attributed to a unique complex sSMC.

Although Brondum‐Nielsen et al. [14] believed that there was no increase of fetal phenotypic abnormalities if the sSMC is inherited from a phenotypically normal parent, this case refutes the belief, as the neonate was born to a healthy non‐consanguineous parents. In addition, phenotypic manifestations in chromosome 2 distal duplication are believed to arise from the distal region of 2q11.2 [15], which is consistent to the finding reported in our case correlated to the observed phenotype contrary to material derived from the proximal part which exhibit no clinical abnormalities.

Al‐Saffar et al. [16] provided a phenotypic description of a patient with pure 2p duplication in the form of dimorphism, esotropia, myopia, left optic nerve hypoplasia, generalized hypotonia with a normal MRI. Although it was derived from a translocation between chromosomes 2 and 13, the normal MRI finding suggests that the nervous system abnormalities may arise from different chromosomes, as significant abnormal MRI findings were found in our case when chromosomes 2 and 15 were involved. It can also be contemplated that genes are dose‐dependent and defects appear when there is a loss of function due to deletion rather than gain of function by duplication [17]. Conversely, Christofolini et al. [18] reported a case of complex sSMC involving chromosomes 15 and 16 in a 6‐year‐old female with dysmorphism and a normal MRI as well. Despite the fact that chromosome15 is less likely to be a culprit in causing an MRI abnormality evident in the previously mentioned reports, Lemskaya et al. [19]. discussed a recent sSMC case involving chromosomes 15 and 9, which had MRI findings similar to our case in the form of corpus callosum dysgenesis with Dandy–Walker variant that was not found in our patient. Hence, central nervous system phenotypic correlation remains genotypically indeterminate in terms of chromosomal derivative origin.

The region (q11.2 → q13.3) in chromosome 15 is known to be a serious breakpoint encoding genetic material responsible for regulation of central nervous system [20]. Several genes have been identified to be located there such as GABAAR, paternal SNRPN and maternal UBE3A genes [21]. The breakpoint identified in our report is more distal suggesting more likelihood of neurological symptoms severity if distal genes are involved.

## Author Contributions

**Yazeed Alayed:** conceptualization, methodology, visualization. **Aziza Mushiba:** supervision, writing – review and editing. **Soha Tashkandi:** data curation, formal analysis, validation. **Yahya Almashham:** validation, writing – review and editing. **Khelad Alsaidi:** formal analysis, investigation. **Abdulrazaq Albohigan:** data curation, formal analysis, visualization. **Amer Jamhawi:** formal analysis, validation. **Mohammad Alkheilewi:** project administration, supervision. **Maryam Alotaibi:** conceptualization, investigation, methodology, writing – original draft.

## Ethics Statement

Approval from the institutional review board (IRB) of King Fahad Medical City (KSMC) was obtained (IRB Registration Number with KACST, KSA: H‐01‐R‐012).

## Consent

Informed consent was also signed by next of kin. Written informed consent was obtained from legally authorized next of kin to publish the details pertaining to the case report of the subject involved. Consent for publication was signed by the next of kind due to subject being a minor.

## Conflicts of Interest

The authors declare no conflicts of interest.

## Data Availability

All data generated or analyzed during this study are included in this published article.
